# Perceptions of the medical relevance of patients` stories of painful and adverse life experiences: a focus group study among Norwegian General Practitioners

**DOI:** 10.1080/17482631.2022.2108560

**Published:** 2022-08-18

**Authors:** Marianne Rønneberg, Bente Prytz Mjølstad, Lotte Hvas, Linn Getz

**Affiliations:** a Tingvoll Healthcare Centre, Tingvoll, Norway; b General Practice Research Unit, Department of Public Health and Nursing, Norwegian University of Science and Technology (NTNU), Trondheim, Norway; c The Research Unit for General Practice and Section of General Practice, Department of Public Health, University of Copenhagen, Copenhagen, Denmark

**Keywords:** General Practice, adverse life experiences, embodiment, causality, dualism, biopsychosocial model, narratives, patient-centred medicine, doctor-patient relationship

## Abstract

**Purpose:**

Adverse life experiences increase the risk of health problems. Little is known about General Practitioners’ (GPs') thoughts, clinical concepts, and work patterns related to eliciting, including, or excluding their patients’ stories of painful and adverse life experiences. We wanted to explore GPs’ perceptions of the medical relevance of stories of painful and adverse life experiences, and to focus on what hinders or facilitates working with such stories.

**Method:**

Eighteen Norwegian GPs participated in three focus group interviews. The interviews were analysed using reflexive thematic analysis.

**Results:**

The participating GPs’ views on the clinical relevance of patients’ painful and adverse experiences varied considerably. Our analysis revealed two distinct stances: a *confident-accepting stance*, and an *ambivalent-conditional stance*. GPs encountered barriers to exploring such stories: scepticism on behalf of the medical discipline; scepticism on behalf of the patients; and, uncertainty regarding how to address stories of painful and adverse experiences in consultations. Work with painful stories was best facilitated when GPs manifested personal openness and prepared availability, within the context of a doctor-patient relationship based on trust.

**Conclusions:**

Clearer processes for handling biographical information and life experiences that affect patients’ health are needed to facilitate the work of primary care physicians.

## 1. Introduction

Listening is considered an essential clinical skill. Competent listening may be particularly important in primary health care, where General Practitioners (GPs) encounter self-selected patients with a wide range of health problems. By listening carefully to the patient, the GP can gather accurate, relevant, and useful clinical information, supporting a shared interpretation or diagnosis on which to base a treatment plan or further action (Launer, 2002; Silverman et al., 2013). However, GPs have no shared knowledge-base focused on the details of whether, when, or how to engage with patients’ stories of painful and adverse life experiences.

During the last decades, a growing body of evidence has demonstrated how subjective experiences (biography) affect health outcomes (biology)—for better and for worse. This subjective turn (Thomasdottir et al., 2015; 2016; Ulvestad, 2018) facilitates the emergence of substantial and constantly evolving scientific knowledge, including previously unrecognized links to potential future health problems—somatic as well as mental—between adverse life experiences in general, and integrity violations in particular. In the wake of previous investigations into so-called psychosomatic diseases, milestone publications pertaining to this topic have come out during the late 1990s and early 2000s. Key examples include: “The Adverse Childhood Experiences Study” (Felitti et al., 1998); “Stress, Adaptation, and Disease” (McEwen, 1998); the introduction of the concept of allostatic overload in stress physiology; and, “Inscribed Bodies” (Kirkengen, 2001), a phenomenological investigation of sexual violation’s impact on health. In “Embodiment: A conceptual glossary for epidemiology” (Krieger, 2005), Epidemiologist Nancy Krieger began using the concept of *embodiment* to accommodate knowledge about the interface between experience and biology. Evidence pertaining to these perspectives has continued to accumulate, supported by increasingly sophisticated methodologies ranging from neuroimaging to epigenetics (Adler & Stewart, 2010; Getz et al., 2011; Halfon et al., 2014; McEwen & Getz, 2013; Teicher et al., 2016). A recent systematic review and meta-analysis examined the financial costs stemming from adverse childhood experiences (ACEs) in 28 European countries. Costs attributed to ACEs were estimated to be 1,1–6,0% of the nations’ gross domestic products (Hughes et al., 2021).

Several questions arise from the above scientific outline. What constitutes a valid theoretical foundation for future primary care (Lynch et al., 2021)? To what extent, and how, should GPs aspire to interact with their patients as whole persons, and relate to the evidence that links biography to biology?

In 1975, Ian McWhinney’s team reported that 22% of 389 Canadian GPs’ patients presented with frankly psychosocial problems. In 14% of the remaining patient consultations, the presenting illness/symptoms seemed to function as a “ticket of admission” to a discussion of more sensitive issues or life problems (Stewart et al., 1975). A large, recent Norwegian study of 1032 Norwegian GPs showed that in 18% of the approximately 20.000 consultations that were evaluated, the doctor had associated the patient’s presenting problem with life strains and stresses. In 5% of the consultations, the GPs were aware of, or suspected, a more explicit association to histories of violence, abuse, or neglect (Johnsen et al., 2020). In other words, GPs across the decades have seemed to acknowledge that psychosocial stress, integrity violations, and other kinds of adversity do affect people’s health and their help-seeking behaviour.

Just how much do GPs actually know about their patients as individual persons? A study among Norwegian GPs published in 1997 found that GPs had very limited knowledge about stressful life conditions and traumatic life experiences of their patients (Gulbrandsen et al., 1997). A more recent study from our own research group confirmed this impression (Mjølstad et al., 2013b). When GPs participating in this study gained new and dramatic biographical information about patients they had known for years, they seemed surprised, and even embarrassed, as though they felt they *should* have known. In cases where they *did* know that a patient had experienced stressful life events, many GPs hardly reflected on how those experiences might have affected the patient’s health (Mjølstad et al., 2013b).

Beyond this, we know little about GPs’ current thinking and customary practices when it comes to listening for, including, or excluding, their patients’ stories of painful and adverse experiences (Box 1). The present project was initiated to increase knowledge about that. Our research questions are: What are GPs’ perceptions of the medical relevance of patients’ stories of painful and adverse life experiences? What hinders or facilitates working with such stories? To our knowledge, these questions have not been investigated before.

**Box 1.** Our understanding of the terminology we use in this paper.

***Biography***: an account of someone’s life, used in a relatively broad sense. ***Story***: a biographical expansion of the term “medical history”, i.e., patients’ statements regarding *events/facts* pertinent to the problem or situation in question. The present study deals with patients’ accounts of existentially painful childhood or adult life experiences, such as having been rejected, betrayed, victimized or abused, be it by someone close or by a stranger, and with patients’ accounts of such *adverse* childhood experiences as war, poverty, neglect, parental criminality or substance abuse, tragic losses of close family members. ***Narrative***: the person’s *subjective understanding and presentation* of a situation and/or a series of events, including their perceptions of their own identity and life expectations. Since a narrative reflects one particular viewpoint, it is open to being reconsidered and changed (Frank, 1998; Launer, 2002). Our study focuses on narratives expressing painful and/or traumatic experiences.

### 1.1. Theoretical perspectives

Our understanding of General Practice/family medicine as a discipline with its own theoretical framework is informed by the writings of Professor of Family Medicine Ian McWhinney. Beyond contributing to the patient-centred method of communication (Levenstein et al., 1986), McWhinney did substantial work as a medical philosopher, describing a comprehensive and holistic (the term *biopsychosocial* might also apply here) theoretical foundation for General Practice/Family Medicine as distinguishable from other medical disciplines (Martin et al., 2014; McWhinney, 1980, 1993; McWhinney & Freeman, 2009). A key characteristic of General Practice, he contended, is that it is shaped by the doctor-patient relationship and the doctor’s evolving knowledge of the patient as a particular person. Following patients over time, experienced GPs tend to develop what McWhinney called an *organismic* mindset (McWhinney, 1996, 2000). *Organismic thinkers* recognize the complex and context-dependent nature of human beings. Referring to Gorovitz and MacIntyre, McWhinney viewed each patient as a unique *particular* someone who, “occupies a region of space, persists through time, has boundaries and has an environment“. He also contended that, “the point about particulars is that their behaviour cannot be explained or predicted solely by applying to them the general laws of science” (McWhinney, 1989, p. 296). A person, like organisms in general, can grow, regenerate, heal, learn, self-organize and self-transcend. Regarding the health impact of adverse experiences, McWhinney wrote: “An organism reacts to the traumas of life as a whole. All significant illness affects the organism at every level, from the molecular to the cognitive and affective” (McWhinney, 2000, p. 137).

Since McWhinney’s time, Evidence Based Medicine (EBM) has dominated the medical discourse, with patient-centred communication remaining a useful communication tool. The call for more genuinely holistic, humane, person-centred medicine (or care) has, however, re-emerged in various milieus and ignited debate (Charon, 2006; Greenhalgh & Hurwitz, 1999; Kirkengen et al., 2012; Launer, 2002; Miles & Mezzich, 2011; Mjølstad, 2015).

## 2. Methods

### 2.1. Approach

To address our questions, we carried out a descriptive qualitative research study among Norwegian General Practitioners. We recognize that such studies do involve researchers’ interpretations (Braun & Clarke, 2021a). This study focuses on experience, in the sense that language reflects reality and is regarded as a tool for communicating thoughts, feelings, and experiences (Braun and Clarke, 2022). It is underpinned by a *critical realist ontology* in which reality is defined as something that exists “out there” while “access to it is always mediated by socio-cultural meanings” (Terry et al., 2017).

Our first reason for choosing focus group interviews to collect data is that they are considered particularly well-suited to studying attitudes and experiences (Kitzinger, 1995), and to exploring fields where little is known in advance (Morgan, 1998). Secondly, we wanted to accommodate the dynamics that focus groups could elicit, with space for a wide range of reflections, including tentative and conflicting views (Kitzinger, 1995). Our approach was inspired by *the semi-structured lifeworld interview* a phenomenological approach that aids in understanding “themes of the lived everyday world from the subjects’ own perspectives” (Brinkmann & Kvale, 2015, p. 27). We developed the interview guide with this in mind, i.e., seeking to explore how GPs understand and subsequently, either work or do not work with patients’ stories of painful and adverse experiences.

### 2.2. Sampling and recruitment

When selecting participants, we purposely recruited GPs who also supervise final year medical students attending in-service training at affiliated healthcare centres. Given the GPs’ clinical teaching and supervising interest and experience, we expected them to be familiar with discussing and reflecting on experiences from their daily work. At the same time, the invited GPs had not been encouraged earlier to think, or to teach, in any specific way regarding the topic in question. Apart from their University affiliation, we assumed that the participants would not differ much from other Norwegian GPs. GP tutors, some of whom had participated earlier in two voluntary courses organized by the university in 2016/17, were invited to participate in this event—focus group interviews. We were not acquainted with the GPs who volunteered to participate, with the exception of brief contacts with some few of them in other professional settings. MR had been one of the lecturers at the first course, but on a topic unrelated to our research topic.

### 2.3. Data collection

The interviews were moderated by MR, as well as by BPM, an established qualitative researcher with experience conducting focus groups. The focus groups lasted 70–90 minutes each. Each focus group consisted of GP tutors of varying ages, from both urban and rural areas. All groups involved both men and women, with a total of 11 men and 7 women participating. Fourteen of the participants were certified as Specialists in General Practice. The GPs represented a total of 303 years of clinical practice, and 147 years of tutoring students (see Table I).

**Table I.** Characteristics of the participating General Practitioners (n = 18).

Participant	Gender	Age	GP Specialist	Years as a GP	Practice location	No. of patients on GP’s list
A	M	32	No	2.5	Urban	900
B	F	76	Yes	31	Rural	1000
C	M	25	No	1	Rural	850
D	F	63	Yes	35	Rural	850
E	M	34	No	4	Rural	900
F	F	69	Yes	37	Urban	1040
G	F	55	Yes	12	Rural	950
H	M	44	Yes	14	Rural	1400
I	M	42	No	3	Urban	1000
J	M	59	Yes	29	Urban	1240
K	F	60	Yes	30	Rural	1150
L	M	60	Yes	5	Urban	900
M	F	50	Yes	9.5	Rural	900
N	M	59	Yes	23	Urban	900
O	M	39	Yes	7	Urban	1250
P	M	62	Yes	32	Urban	1000
Q	F	42	Yes	11	Urban	1000
R	M	64	Yes	17	Rural	600

In all three interviews, the moderators set the scene by recounting an authentic, anonymized patient narrative involving adverse childhood experiences, excerpted here:

‘Anna’ was stigmatized and severely harassed during childhood for being the illegitimate daughter of an enemy soldier. As an adult, she developed many health problems.

Next, the moderators summarized an epidemiological study showing a dose-response association between self-perceived childhood difficulties and multimorbidity later in life (Tomasdottir et al., 2015). The group participants were then invited to respond, openly, and spontaneously, to that story and/or to share similar stories from their own practices.

Subsequently, the moderators applied a semi-structured interview guide comprised of flexible, open-ended questions (see supplementary material). All interview audio was recorded and transcribed, verbatim, by MR, who was also responsible for making field/reflection notes after each session.

During the interviews, the moderators paid particular attention both to *complementary interactions*, those exchanges in which participants supported and enhanced each other’s views and experiences, and to as *argumentative interactions*, exchanges in which participants questioned one another or openly expressed disagreement (Kitzinger, 1994).

### 2.4. Data analysis

We applied *reflexive thematic analysis* to our data (Braun & Clarke, 2019 and 2022; Terry et al., 2017). Generally, such thematic analyses seek to reveal patterns of meaning across the data (Braun and Clarke, 2020). Typically, codes and themes are developed inductively (Terry et al., 2017). Reflexive thematic analysis is well-suited to analysing data derived from research questions related to people’s experiences and perceptions, and it can be applied to heterogenous samples (Braun & Clarke, 2021b).

All four authors participated in the analysis of the data, following the six analytic steps described by Braun and Clarke (Braun & Clarke, 2006). First, we familiarized ourselves individually with the data while making observational notes. In the next step, we compared and discussed our observations and notes. Codes were then generated inductively, informed by the research questions. While most codes were descriptive (semantic) some were interpretative (latent). From the themes that we identified based on these codes, we formulated potentially overarching themes. We complied thematic maps to keep track of both codes and candidate themes. We completed the analysis by returning to the transcripts to check that the identified themes did represent the most relevant subjects discussed during the interviews. We applied McWhinney’s theoretical framework as *a substantive theory* to expand our findings and to sharpen our analytic focus (Malterud, 2016).

### 2.5. Ethical considerations

Before conducting the focus group interviews, we contacted the Regional Committee for Medical and Health Research Ethics (REK Midt) and described the study. No formal application was required by them, nor by the Norwegian Centre for Research Data (NSD), with whom we also consulted. Written consent to participate in the interviews/analyses was obtained from all focus group participants. The focus group audio recordings and transcripts have been secured in accordance with Norwegian research regulations.

## 3. Results

Our analysis of the three GP focus group interviews revealed two main stances regarding the medical relevance of patients’ stories of painful and adverse experiences. The first position was characterized by explicit acknowledgement of the medical relevance of such stories. We term this the *confident-accepting stance*. GPs taking this stance were found in all three groups, often among those with the most clinical experience. They were few in number, however. For a variety of reasons, the majority of the participants, both novel and experienced GPs, took the second position, characterized by an *ambivalent*and only *conditional* acceptance of addressing patients’ stories of painful and adverse experiences. They expressed concerns related to questions regarding the medical relevance of such stories, as well as to whether GPs should be addressing them at all.

These two positions—*confident-accepting*, and *ambivalent-conditional*—were not sharply demarcated but were rather the poles of a continuum. During parts of the discussion, several of the GPs’ views oscillated between the stances. However, one or two of the more experienced GPs in each focus group maintained a *confident-accepting stance* throughout the entire discussion.

In addition to revealing these two stances regarding GPs’ relationship to patients’ stories of painful and adverse experiences in their clinical practices, our analysis identified factors hindering or facilitating their relating to such stories. As barriers, we identified scepticism on behalf of the medical discipline and on behalf of the patients. Many GPs wondered whether work with patients’ stories of painful and adverse experiences belongs within the scope of General Practice. Most participants were under the impression that many patients do not want to tell their GPs such stories; some seemed to imply that GPs might feel at a loss when faced with patients` painful stories.

On the other hand, certain qualities of the GP as a person were likely to facilitate the doctors in addressing relevant stories, including the GP being willing and able to allocate sufficient time to listen, within a context of a doctor-patient relationship based on trust.

### 3.1. The confident-accepting stance

As outlined above, some GPs in all three focus groups immediately took, and maintained, a confident-accepting stance in relation to working with patients’ stories of pain and adversity.

These confident-accepting GPs often validated the relevance of the vignette by recounting similar stories from their own practices, as one of them said in response to the opening vignette: “I know a lot of Annas.” (GP F)

In the discussions that followed, these GPs seemed to take the biography/biology interconnectedness as a given and rarely referred to scientific studies or theoretical frameworks to support their view. Their responses seemed primarily experience-based and case-oriented; they included types of adversity extending beyond the explicit, categorizable events or trauma typically discussed in the medical literature. For instance, they would agree that having a “longstanding lack of feeling safe and protected” would generally increase people’s susceptibility to disease. The confident-accepting GPs hardly distinguished between health problems that are classified conventionally into categories of somatic vs. psychiatric, or medically acknowledged vs. medically unexplained. For instance, GP Q associated a patient’s childhood history of being severely bullied and socially excluded with both muscular pain and lung disease in adult life:

I have a patient in my practice who’s in an almost identical situation – she was also called *‘that German kid’* and she suffers from asthma and fibromyalgia, too. (GP Q)

To explain how they reasoned and worked, some of the confident-accepting GPs turned to everyday *language metaphors*. GP J referred to “good ballast” to explain how growing up in a safe and supportive environment tends to make people resilient. GP L referred to adverse life experiences as the patient’s “backpack” and that the GP might help the person “re-pack”. GP F stated that her most important role was to listen to patients’ stories, even repeatedly, so that they could “air themselves out”. Secondly, she should serve a supportive role for the patient, as a metaphorical “walking stick”. Unresolved ethical and practical issues restrain health professionals from entering stories of painful or adverse experiences into patients’ medical records, yet GPs often remembered them. One GP referred to this undocumented knowledge as “the shadow files”.

The consultation style of the confident-accepting GPs typically involved thinking through and applying a flexible strategy when it seemed that the patient’s painful story might have specific medical relevance. For example:

We have to be a bit careful. People are so different. Some are interested in learning about the causes of their ailments – others aren’t. So, I think of it as an issue of autonomy. The patient has to be the one who’s in charge here. The GP can ask some questions, and sometimes the answer won’t come until a later visit, after the patient has given it some thought – after it has sort of *‘ripened’*. (GP L)

### 3.2. The ambivalent-conditional stance

This stance is characterized by an *ambivalent* response to the concept that life experiences and health are interconnected, and a *conditional* acceptance of patients` stories of painful and adverse experiences being relevant medically and falling within the GP’s mandate to explore.

Responding to the introductory vignette, some of these GPs seemed reluctant to acknowledge any general, biomedically relevant connection between a difficult childhood and adult disease, as one exclaimed: “Generally speaking, I’m sceptical about these studies or results.” (GP R)

These GPs had doubts as to how such childhood experiences could cause somatic diseases. GP E, for example, was sceptical about the existence of a link between stressful life events and what he referred to as “real” somatic diseases:

Well, I think some of the symptoms in the story presented in the vignette could probably be due to childhood difficulties, but other diseases, like vasculitis and recurring pneumonia … I find it hard … I couldn’t say that those are the results of a difficult childhood. (GP E)

GP E’s statement exemplifies the tendency among those taking the ambivalent-conditional stance to draw a clear line between somatic and mental diseases/disorders. While they did not link somatic diseases to experienced adversity, they frequently associated mental problems and so-called “medically unexplained physical symptoms” (MUPS) with adverse life experiences. They often referred to symptoms that might have alerted them to a history of adversity as “diffuse”, “non-specific”, and/or “subjective”, effectively placing them in a diagnostic no-man’s land, some stressing how uniquely personal and unpredictable such linkages would be. Nonetheless, while concluding that the eliciting of such stories was of limited clinical value, they seemed eager to share examples of patients who were in good health—despite considerable adversity earlier in life.

A distinct feature of the ambivalent-conditional stance was that these GPs appeared willing to work with stories of adversity if and only if the patient explicitly asked them to do so, and if they considered the patient capable of exploring and carrying out necessary life changes. Such a *conditional willingness* to include patients’ stories in clinical work can be exemplified by the following exchange between two GPs taking the ambivalent-conditional stance (GP H and GP J), and one GP who held a confident-accepting stance (GP F). The three discussed allowing a patient to keep repeating what one GP perceived as, “Just the same story over and over again”. Was that a waste of time or might it be beneficial in some way?

GP H: But if [the patients] keep coming [to see their GPs] just to talk about the same things, I don’t think that’s really useful.

GP J: I pretty much agree. We have to look for some action that could create change. That’s what’s interesting: How to get them to stop gnawing on the same old bone all the time. Help them get onto something different. Move on.

The GP who generally took the confident-accepting stance responded:

GP F: I do have faith in repetition […] I think many keep the same stories going, and use their regular GP as an outlet, a safety valve. But, I think we ought to limit our ambitions sometimes to serving as somebody’s crutch, so they can manage to live with their problems.

### 3.3. Barriers to working with patients’ stories

#### 3.3.1. GPs’ scepticism on behalf of the medical discipline (as mandated by society)

Some GPs in all three focus groups expressed uncertainty as to whether work with painful and adverse experiences fits into the scope or mandate of a busy GP’s clinical practice, irrespective of the stories’ potential medical relevance:

[Working with stories of painful and adverse experiences] doesn’t fit with my daily routines as a GP. Problems have to be solved then and there. (GP R)

Well, I’m a GP. I’d have referred [that patient] to a specialist. (GP B)

These quotations illustrate how both time constraints and presumptions of what ought to be expected of a doctor represent obstacles to including work with patients` stories of painful and adverse experiences in their practice.

During discussions about clinical suitability, there were participants in all three groups who indicated that it might actually be up to the GP to develop a consultation style that either encourages patients to share painful stories or discourages them from doing so. For instance, GP K described the self-protective strategy of assuming a formal and highly structured consultation style, particularly when encountering problems that might be rooted in adverse experiences:

Well, I don’t have many of these patients with behavioural problems or drug addiction on my list. I suppose my being so overtly structured helps me manage to avoid some types of patients. (GP K)

#### 3.3.2. GPs’ scepticism on behalf of the patients

Several GPs argued that the best solution for patients with a history of painful and adverse experiences would often be to simply *leave the painful stories behind*. The best clinical approach, these GPs agreed, is to place the focus on the patients’ strong sides and resources. For example:

Whenever patients bring up something sad, we try to stick to all the good things that have happened, to hold onto whatever is positive. (GP R)

GPs who shared this view were also among those finding it inappropriate for the GP to take the initiative in bringing up painful stories but rather leave it up to the patient.

Some scepticism on behalf of the patients focused on the *right to confidentiality*. The content of painful stories may be highly sensitive, and patients may simply not want to share them with their GP, irrespective of potential medical relevance.

Some GPs told of patients who had rejected an invitation to explore connections between their life experiences and their presenting complaints. Such refusals, they reasoned, might indicate the patient’s lack of insight into the existence, and thus the potential impact, of such connections. Once an attempt to elicit such stories had been rejected, the GPs rarely repeated the suggestion. Those who still wished to try expressed having difficulty devising alternative ways to open the topic.

#### 3.3.3. How to do it? Feeling at a loss

Despite the professional barriers described above, most GPs did acknowledge having encountered patients whose adverse life experiences they suspected were relevant to the patients’ current health problems. Several GPs reported feeling at a loss in such circumstances. As one put it:

Sometimes, even if I have the feeling that there’s something there … [that that patient has a story] that I really want to ask about, to learn about … still, I struggle to figure out how to ask. I find myself thinking, ‘No, this isn’t the right time.’ I’m afraid it might take too long, like I’m about to open a wasp’s nest. I’m afraid I might retraumatize my patient … or hurt the person’s feelings if my questions seem inappropriate, or insulting … (GP A)

This highlights several of the obstacles some GPs mentioned facing. First, they felt uncertain about how to position themselves to ask specifically about, and relate professionally to, painful stories. Second, they worried they might *do more harm than good* by encouraging patients to bring such stories to the surface, even when the person might appear ready to share them. Third, the quote above also indicates the tentative, vague way the GPs spoke as they struggled with whether, how, and when to address patient stories of pain and adversity. They made frequent use of qualifying phrases such as *maybe … one might think that … it’s hard to tell if*, as if grappling to find the right words.

Several GPs said it *takes courage* to ask patients pointedly about adverse life experiences, and that a *lack of courage* may sometimes have kept them from asking, even when they suspected that what a patient might have endured had medical relevance. One GP shared, however, that the clinical experience in his professional role as doctor had had the opposite effect, so that it became easier to elicit stories of painful and adverse experiences:

Being a doctor means you can ask about anything – if you ask respectfully, that is. And, gradually, I’ve become braver. (GP L)

### 3.4 Pathways to eliciting medically relevant stories

Despite the continuum of stances in relation to patients’ narratives of painful and adverse experiences, all participating GPs shared some ideas on factors that might facilitate GPs, and others, in working with them.

#### 3.4.1. The cornerstone: a doctor-patient relationship based on trust

The consensus reached among participants in all three focus groups was that a GPs' work with stories of adversity required a quality clinical relationship, with a solid doctor-patient relationship based on trust paving the way. Some patients seemed simply to know that they could show up for their regular doctor appointment and share their stories spontaneously, making it unnecessary for the GP to even ask. Knowing patients over time also helped GPs notice and then comment, carefully and appropriately, on changes in patients’ behaviour or habits. Quite often the patient would respond to such comments by sharing a story about a painful experience:

I knew that a certain [patient] really liked to take trips up to his cabin. One day, he suddenly said, ‘Well, I haven`t been up at my cabin very much this fall.’ So, I said, ‘Okay.’ And then I asked, ‘But why not?’ That question started a stream of revelations. (GP Q)

#### 3.4.2. A GP who is open and ready

Participants in all three focus groups considered the patients’ perception of the doctors’ personal qualities and attitudes during clinical encounters to have an impact on whether or not patients would share their painful stories. Pivotal factors included how trustworthy physicians seemed, how willing they were to listen, and, generally, how empathetic they seemed to be.

[Patients] have to feel that they can trust their doctor … You [the GP] have to signal that you have time to listen, no matter what … I’ve had people come to see me about things they haven`t shared with anybody, ever. To me, that’s a clear declaration of trust. (GP J)

GPs also noted that simply asking the (presumably) right questions was not always enough to encourage patients to share their inner pain. Their own timing had to be right as well. Two GPs pointed out that experiences in their own lives, the mood they might have been in on a particular day, or perhaps even non-verbal cues, might have helped alert them to medically relevant stories, and also aided patients in sharing them. As one GP put it:

My colleagues and I have sometimes noticed at the end of a certain day that several of the patients we’d seen had started crying. It`s a bit strange. I wonder if we GPs have times when we’re more empathetic, more open to people’s pain and what they struggle with, and that makes it easier for our patients to bring up whatever is burdening them. (GP D)

After having listened to the group discuss which personal qualities might be valued by patients who felt the need to talk to their doctors about adverse experiences, one previously sceptical GP commented:

I believe we can all be supportive when we work with such issues. I really do. (GP R)

#### 3.4.3. Time is magic

Time and time management were brought up by several GPs in relation to their work with patients’ stories of adversity. There was nearly unanimous agreement about the importance of having enough time to listen, and being able to signal to the patient, verbally and/or non-verbally, that there was time available. As one GP put it, enthusiastically:

You’ve simply got to have enough time. In my experience, that’s when magic happens. (GP O)

Some GPs said they could sometimes organize their appointment schedules to leave extra time for when they expected it would be needed. At other times, however, they simply had to make room in the middle of a busy day for an unexpectedly lengthy consultation. GPs also noted how sensitive some patients were to the GP having spent more time than might have been necessary; some patients even apologized if their consultations had lasted longer than usual. These GPs reassured their patients that the extra time had been well spent and useful.

## 4. Discussion

### 4.1. Main findings

Our study documents the reflections of 18 University-affiliated, GP supervisors in Norway, on what hinders or facilitates GPs’ work with patients’ stories of painful and adverse life experiences. The most striking finding was the diversity in how the GPs regard the medical relevance of working with their patients` stories of such experiences—ranging from a completely confident and proactive stance to a deeply ambivalent and conditional stance.

Another notable feature was the way the GPs spoke when discussing their reflections and experiences with patients` stories: the frequency of verbal hesitations was high, as were tentative and incomplete utterances, and the use of metaphors. We connect this to a lack of role models that leaves them unfamiliar with appropriate terminology, limiting their ability to describe their experiences, interpretations, and viewpoints regarding their work with patients’ stories and narratives in a concise, confident, and professional language. Ian McWhinney describes a similar phenomenon in his paper, “The Meaning of Holistic Medicine”. He gives an account of how physicians who try to work in a holistic manner and take patients’ life experiences and lifeworld into account, struggle to explain how they work: “He finds it much easier to practice the method than to articulate it, as there is no readily available taxonomic vocabulary, as there is with the conventional school” (McWhinney, 1980, p. 1096). His term, “conventional school” apparently refers to mainstream medical thought and practice, often termed “biomedicine”.

To our surprise, not one of the participating GPs mentioned that such stories evoked, or might evoke, memories of similar experiences in their own histories, or that this kind of triggering might represent an unnamed obstacle to working with patients’ painful stories. It would be valuable to delve into the meaning and consequences of this conspicuously absent content.

### 4.2. Theory of science: causality and dualism

The GPs’ two main stances in relation to stories of adversity might be seen as being rooted in two differing views of what to consider as trustworthy medical knowledge. GPs who took the confident-accepting stance to painful stories often referred to what they themselves had observed and learned from countless encounters with patients. In other words, their accumulated experience created a base of *tacit* knowledge (Polanyi & Sen, 2009) and practical, clinical wisdom (Malterud, 1995). GPs who took the ambivalent-conditional stance to patients’ stories based their reasoning more exclusively on *formal* medical knowledge. In response to the introductory vignette’s suggestion of how life experiences might impact health, their reluctance may be linked to the fact that documentation of the interconnectedness between life experiences and health is still treated as more or less separate from conventional, biomedical thinking (Karunamuni et al., 2021; Kirkengen et al., 2016).

GPs used tentative language during the focus group discussions, regardless of the stance they took. It seems appropriate here to posit that both the GPs’ differing stances in relation to stories of adversity and their tentative speech patterns are associated with indefinite and competing *understandings of causality* within medicine (Cartwright, 2011; Worrall, 2010). As outlined by philosophers in the CauseHealth Network, modern medicine is characterized by a marked tension between differing understandings of evidence and causality. Evidence-Based Medicine and Person-Centred Healthcare represent two different perspectives on reality, i.e., diverging ontologies (Anjum, 2016). EBM is founded on empiricism and abstractions, favouring observable, group-based data, predictability, and a *regularity theory of causation*. In contrast, Person-Centred Healthcare emphasizes complex and context-dependent, individual pathways, based on a theory of *causal dispositionalism* (Anjum, 2016; Anjum et al., 2020), in line with McWhinney’s *organismic thinking* (McWhinney, 1996, 2000). Both approaches are medically relevant but not directly compatible. In addition to encountering different perspectives on causation, most doctors have been trained in a healthcare system strongly influenced by psyche/soma dualism (Davidsen et al., 2016). Neither of the topics of causality or dualism was raised explicitly in our interviews, but we believe the GPs’ differing stances make sense when seen as expressions of implicitly differing perceptions of holism, dualism, and causality. The experienced GPs who took a confident-accepting stance to stories of adversity seem to have supplemented their biomedical health perspectives with *organismic thinking*, while the more sceptical GPs remained within a more conventional, biomedical perspective, in line with EBM. Nonetheless, the confident-accepting stance in relation to painful stories did not strike us as incompatible with EBM. The GP’s statement in response to the introductory vignette, “I know a lot of Annas”, is relevant, both from the perspective of regularity, i.e., that longstanding marginalization is, in general, a risk factor for health problems, and from the perspective of causal dispositionalism, i.e., emphasizing that the particular person’s story has unique features and potentials.

### 4.3. What to pick up on, and what to do with it

We noted that GPs identified various barriers to working with patients’ stories of painful and adverse experiences. One was their explicitly stated concern that probing into stories about adversity might have significant negative effects, either immediately if patients were to feel invaded, or in the long run if it were found that a medical focus on painful experiences did more harm than good. We found it interesting that when Ian McWhinney encountered similar concerns in 1980 (McWhinney, 1980), he interpreted them as expressions of two common and crucial misconceptions regarding “holistic” General Practice. The first was the idea that holistic GPs pry into patients’ private lives. McWhinney countered that a good GP gradually develops an understanding of the patient through careful observation and listening, thus avoiding premature or invasive inquiries. The second misconception that McWhinney disputed was that sensitivity to people’s life stories in a medical context might contribute to the “medicalization of life”. This contention, he warned, has the potential to hamper effective clinical practice; it is often clinically impossible to draw a meaningful distinction between problems of life and biomedical illness (McWhinney, 1980).

A recent Danish study of patients with multimorbidity who visited their GPs on a regular basis, sheds light on some of our findings (Joensson et al., 2020). The study documents what some of our participating GPs had sensed: that some patients do prefer to keep sensitive information from their GPs, based either on an explicit wish for privacy or on the fear of seeming inferior in some way. Moreover, also with direct relevance to our findings, patients tended to make judgements regarding what type of information might be welcome in a clinical encounter, i.e., worthy of a doctor’s attention. The authors of the Danish study conclude that patients’ fear of bringing up something medically irrelevant might result in the omitting of information that might have helped the practitioner arrive at a better understanding of patients’ health problems. Other studies support the premise that increased medical attention to the patient’s lifeworld in primary care does facilitate more humane and effective treatment and lead to better outcomes; those authors also assert that doctors can be sensitized to deliver such care (Barry et al., 2001; Gulbrandsen et al., 1997). However, clinicians may find it uncomfortable to explore patients’ painful and adverse experiences, particularly if they are uncertain whether such work ought to be part of a GP’s professional repertoire at all.

Implicit in overt expressions of scepticism—including statements such as, “it takes courage”—may be the fear of addressing the topic in so awkward and unproductive a manner that it would not help the patient. It may also imply an underlying inclination among some GPs to avoid all such encounters with unresolved stories of personal pain. The noteworthy absence of GPs’ self-referential associations to patients’ painful stories may further support that interpretation.

### 4.4. Reflexivity, strengths and limitations

The focus group interviews were jointly planned and conducted by two experienced GPs (MR and BPM), one of whom is an established, qualitative researcher (BPM). MR has worked as a GP in a rural area for 15 years and is also a GP tutor. LG practiced earlier as a GP and is now a Professor of Behavioural Sciences. LH has worked for decades as a GP in Denmark and is also an experienced qualitative researcher. As professionals, the authors share their recurring recognition that patients’ past and current medical problems and their stories of painful and adverse experiences are related. LG, BPM and LH have also conducted research into that subject.

As moderators of the focus groups (MR and BPM), we emphasized that the group members be allowed to speak as freely as possible. Questions were open-ended, and we maintained awareness of the importance of setting aside our own preconceptions. Our field notes, which we wrote immediately after each interview, included participants’ non-verbal responses. We also described responses we found especially interesting or surprising, such as those that showed apparent ambivalence or hesitancy. We paid particular attention to such aspects of our data when analysing the material.

The author groups’ *insider perspective* facilitated the rapid establishment of collegial contact with the participants and thus their ensuing discussions (Dwyer & Buckle, 2009). On the other hand, the situatedness and presuppositions shared by researchers and participants alike may have blinded us to perspectives that researchers with other backgrounds might have focused on and probed.

We do not know whether interviewing GPs who were not also University-affiliated tutors of medical students might have impacted our results. At the same time, other than the common denominator of being GP tutors, the focus groups were heterogenous, for example, regarding age, gender, years of experience.This would seem to indicate that our results might be transferable to other GPs. Also, our having elicited and identified an unexpected and notable diversity of viewpoints and working habits within what might well be presumed to be a resourceful subgroup of GPs, does suggest that our findings have high validity and relevance. The fact that several participants expressed uncertainty to the concept of medical relevance as early as our presentation of the opening vignette, suggests that they were not tailoring their reactions to try to please us, and that the subsequent analysis has not been unduly influenced by our own preconceptions.

We applied principles of *information power* (Malterud et al., 2019) to guide the sample size of our study. The relatively narrow aims of the study, the purposive sampling strategy, robust dialogues during the focus group interviews, and the diversity of participants’ perspectives, would suggest that the data *information power* is sufficient.

To ensure rigour and transparency, we consulted Braun and Clark’s twenty guiding questions to assess our thematic analysis research quality (Braun & Clarke, 2021a). We made sure that the criteria for questions 1,2,3,4,5,6,9,10,11,13,14 and 17 were met, and that potential pitfalls regarding questions 7,8,12,15,16,18,19 and 20 were avoided.

## 5. Conclusions and future implications

Our study identified a wide diversity of views among GPs regarding the medical relevance of patients’ stories of pain and adversity. Many participants seemed frustrated, even bewildered, when shifting between conventional, abstract biomedical knowledge and a more holistic, person-centred approach to individual patients.

It is not only inefficient but also unethical to leave it to individual doctors and patients to manoeuvre within a medical paradigm characterized not only by incomplete knowledge but also by unresolved theoretical tensions. These can render both parties deeply uncertain about what is relevant to tell, to ask, and to question (Assing Hvidt et al., 2017).

As GP academics, we believe more space should be allocated for biographic information and life experiences that may affect patients’ health. In the meantime, if patients’ stories of painful and adverse experiences are to be integrated into GPs’ practices, clearer processes for handling biographical information, including the life experiences that impact on patients’ health, need to be developed. This calls for knowledge about the interconnectedness between life experiences and health to be integrated into medical curricula. Instruction and guidance in how to work with patients’ stories of painful and adverse experiences in a safe and professional manner are also imperative. Providing care that takes the whole person into consideration has been shown to increase patient satisfaction and to reduce costs (Weiner & Schwartz, 2015). Thus, a skilled and confidently nuanced approach to patients’ life stories is likely to benefit not only patients but also their doctors (Frank 1998; Gronseth et al., 2020).

## Supplementary Material

Supplemental Material
