# Supplementary material for: Perceptions of the medical relevance of patients` stories of painful and adverse life experiences: a focus group study among Norwegian General Practitioners
Source: Int J Qual Stud Health Well-being. 2022 Aug 18;17(1):2108560. doi: 10.1080/17482631.2022.2108560 (PMC9397424; doi:10.1080/17482631.2022.2108560)
Supplement: Supplemental Material [file ZQHW_A_2108560_SM4213.docx]

**SUPPLEMENTARY MATERIAL**

**Interview guide**

1. Now that you have heard this story (the vignette about Ann); what are your thoughts? Do you have any such patients?
2. As a GP, how do you gain access to patients’ stories? (In what situations do you learn about patients’ stories? In what situations do you specifically ask about patients’ stories?)

1. Is knowledge about patients’ stories relevant for us in our work as GPs?

If yes, how? (Examples / specific cases)

If no, why not?

1. Have you ever been in a situation in which you sensed that the patient might have a story that would be relevant, would help you understand his/her symptoms? What characterized such situations? (provide examples/ specific cases)

When you sensed such a story, how did you attempt to reveal it?

What types of patient’s stories would you consider particularly relevant?

1. Some patients talk about many things – large and small. How can a doctor tell that a particular story from a patient’s life might be medically relevant?

What features signal that an aspect of a patient’s life history is significant?

1. How does knowledge of patients’ stories affect you as GPs? Have you ever experienced that insight into patients’ stories has impacted the doctor-patient relationship? If so, how?
